# Left ventricular strain and strain rate by 2D speckle tracking in chronic thromboembolic pulmonary hypertension before and after pulmonary thromboendarterectomy

**DOI:** 10.1186/1476-7120-8-43

**Published:** 2010-09-27

**Authors:** Nicholas Olson, Jason P Brown, Andrew M Kahn, William R Auger, Michael M Madani, Thomas J Waltman, Daniel G Blanchard

**Affiliations:** 1 Division of Cardiology, University of California San Diego Medical Center, 200 W. Arbor Drive, San Diego, CA 92103, USA; 2 Division of Cardiology, UCSD Sulpizio Family Cardiovascular Center, 9350 Campus Point Drive, La Jolla, CA 92037, USA; 3 Division of Pulmonary Medicine, UCSD Sulpizio Family Cardiovascular Center, 9350 Campus Point Drive, La Jolla, CA 92037, USA; 4 Division of Cardiothoracic Surgery, UCSD Sulpizio Family Cardiovascular Center, 9350 Campus Point Drive, La Jolla, CA 92037, USA

## Abstract

**Background:**

Echocardiographic evaluation of left ventricular (LV) strain and strain rate (SR) by 2D speckle tracking may be useful tools to assess chronic thromboembolic pulmonary hypertension (CTEPH) severity as well as response to successful pulmonary thromboendarterectomy (PTE).

**Methods:**

We evaluated 30 patients with CTEPH before and after PTE using 2D speckle tracking measurements of LV radial and circumferential strain and SR in the short axis, and correlated the data with right heart catheterization (RHC).

**Results:**

PTE resulted in a decrease in mean PA pressure (44 ± 15 to 29 ± 9 mmHg), decrease in PVR (950 ± 550 to 31 ± 160 [dyne-sec]/cm^5^), and an increase in cardiac output (3.9 ± 1.0 to 5.0 ± 1.0 L/min, p < 0.001 for all). Circumferential and posterior wall radial strain changed by -11% and +15% respectively (p < 0.001 for both). Circumferential SR and posterior wall radial SR changed by -7% and 6% after PTE. While the increase in posterior wall SR with PTE reached statistical significance (p = 0.04) circumferential SR did not (p = 0.07). In addition, septal radial strain and SR did not change significantly after PTE (p = 0.1 and 0.8 respectively). Linear regression analyses of circumferential and posterior wall radial strain and SR revealed little correlation between strain/SR measurements and PVR, mean PA pressure, or cardiac output. However, *change *in circumferential strain and *change *in posterior wall radial strain correlated moderately well with changes in PVR, mean PA pressure and cardiac output (r = 0.69, 0.76, and 0.51 for circumferential strain [p < 0.001 for all] and r = 0.7, 0.7, 0.45 for posterior wall radial strain [p = 0.001, 0.001, and 0.02, respectively]).

**Conclusions:**

LV circumferential and posterior wall radial strain change after relief of pulmonary arterial obstruction in patients with CTEPH, and these improvements occur rapidly. These changes in LV strain may reflect effects from improved LV diastolic filling, and may be useful non-invasive markers of successful PTE.

## Introduction

Once thought of as a rare complication, chronic thromboembolic pulmonary hypertension (CTEPH) has recently been demonstrated to be relatively common in the post-pulmonary embolism patient. While most epidemiological studies place the incidence at approximately 1 percent, a recent study estimated the incidence at 3.8% [1-4]. CTEPH results from the organization of a pulmonary embolus with extensive intimal hyperplasia of the adjacent pulmonary vasculature [5,6] The resulting increase in pulmonary vascular resistance leads to severe right ventricular enlargement and overload. In the absence of coexisting cardiomyopathy or systemic hypertension, left ventricular (LV) systolic function remains normal [7]. However, left ventricular chamber distortion (decreased LV end-diastolic volume and abnormal eccentricity index) and abnormal diastolic filling (E/A reversal, systolic-dominant pulmonary venous flow) are common and have been proposed as major pathophysiologic mechanisms for impaired cardiac output in this population [8]. The chamber distortion and abnormal diastolic parameters improve rapidly following pulmonary thromboendarterectomy (PTE) [9-11]. Therefore, echocardiographic evaluation of the left ventricle may be a useful tool to assess CTEPH severity and response to PTE. Two-dimensional (2D) speckle tracking is a novel way to quantify LV global and regional function via strain and strain rate (SR) analysis [12]. We evaluated 2D speckle tracking measurements of radial and circumferential strain and SR in the LV short axis, and correlated the data with right heart catheterization results to assess whether strain and SR are useful parameters of LV function in CTEPH before and after PTE.

## Methods

### Patients

The pre- and post- PTE 2D echocardiograms of sixty consecutive patients with CTEPH who underwent PTE were analyzed by 2D speckle tracking. Thirty patients demonstrated adequate 2D speckle tracking, and were studied 7.4 ± 4.7 days before and 9.2 ± 2.6 days after PTE. Adequacy of 2D speckle tracking was determined during strain and SR analysis of the 2D grayscale images with the EchoPac 6.1 software as outlined in the "Speckle Tracking Analysis" section below. Only echocardiographic studies that yielded "good" tracking in all segments were used in the analysis. Right heart catheterizations (RHC) were also performed 6 ± 5 days before and in the operating room immediately after completion of the PTE. Pulmonary artery pressure (PAP), pulmonary vascular resistance (PVR), central venous pressure (CVP), and cardiac output (CO) were determined. All PTE surgeries were performed between January 2006 and September 2008 at UCSD Medical Center according to standard techniques at our institution described previously [13]. The research protocol for this study was approved by the UCSD institutional research review committee.

### Echocardiographic Examination

The echocardiographic examination was performed using a Vivid 7 cardiovascular ultrasound system (GE VingMed, Horten, Norway). Examinations included measurements of the LV end-systolic and end-diastolic diameters. Ejection fraction (EF) was calculated using the biplane modified Simpson method. Fractional shortening was measured in the parasternal long axis. All measurements were made in accordance with the recommendations of the American Society of Echocardiography [14].

### Speckle Tracking Analysis

**Figure 1:**
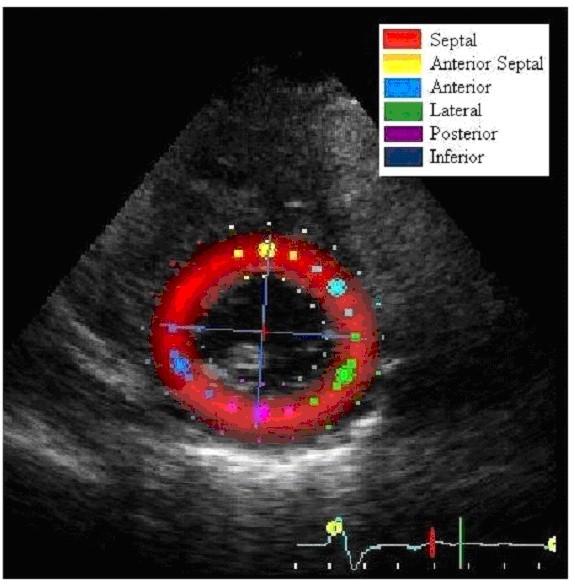
**For 2D speckle tracking, a R.O.I. was selected at the level of the papillary muscles**. The myocardium was divided into 6 segments in accordance with the standard 16 segment left ventricular model.

Standard grayscale 2D images were acquired in the parasternal short-axis view at the level of the LV papillary muscles. The myocardium was divided into 6 segments according to the standard 16 segment left ventricular model as displayed in Figure 1[15]. All of the images were recorded with a frame rate of at least 30 fps to allow for reliable operation of the software (EchoPac 6.1). From an end-systolic single frame, a region of interest (ROI) was manually mapped to the endocardial border and its width was adjusted to span the entire myocardium (Figure 1). The automated tracking algorithm followed the myocardial interference pattern (speckle pattern) from this single frame throughout the cardiac cycle. This tracking algorithm additionally reported how well speckle tracking was proceeding from frame to frame over the cardiac cycle. Each individual myocardial segment (Figure 1) was analyzed independently and labeled as poor, average, or good tracking. Further manual adjustment of the ROI was performed if needed to obtain optimal tracking. Only images that demonstrated good tracking in all myocardial segments in both their pre- and post-PTE echocardiograms were used in the analysis.

**Figure 2:**
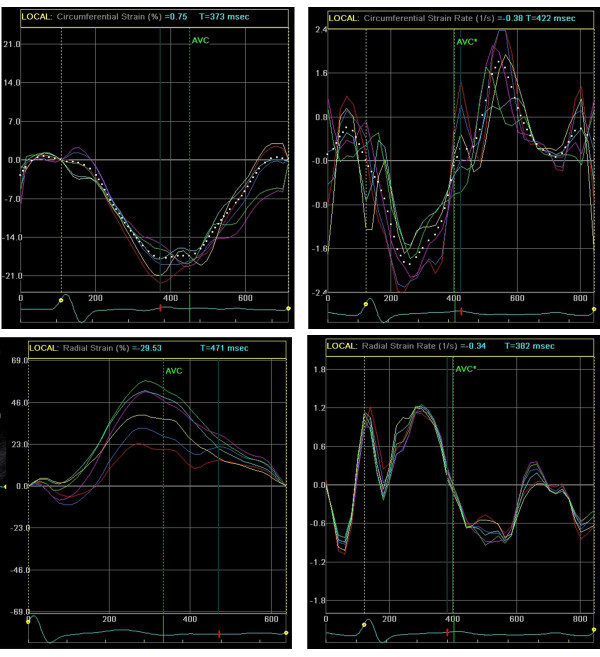
**Left ventricular global circumferential strain (top left, dotted) and strain rate (top right, dotted) as well as radial strain (bottom left) and strain rate (bottom right) are displayed from a patient with CTEPH**. The septal and posterior walls are outlined in red and purple respectively. The posterior wall radial strain is displayed post-PTE demonstrating the relative the hypokinesis of the septal segment (red).

Global circumferential strain and strain rate, which represents an average of all 6 segments (Figure 2, upper, dotted), was used in our analysis. For radial strain and strain rate the posterior wall (purple) and septal wall (red) were used in our analysis. We chose these two segments for analysis because one (septal) is directly affected by the right ventricle (RV) while the other (posterior) is most removed from the RV and should be relatively unaffected. Strain and strain rate measures were plotted against time in the EchoPac 6.1 output screens displayed in Figure 2.

### Statistical Analysis

The variables in our study (strain and SR) were dependent (i.e., each post-PTE value needed to be compared to its corresponding pre-PTE value for significance). Therefore, a paired two-tailed Student's t-test was utilized to determine differences in strain and SR following PTE. 95% confidence intervals for changes in strain and SR as well as p values were determined. Plots of strain vs. RHC parameters and change in strain vs. change in right heart catheterization (RHC) parameters were analyzed with linear regression software http://www.Wessa.net[16]. Linear regression analyses of circumferential strain and posterior wall radial strain vs. RHC data (PVR, mean PA pressure and cardiac output) were performed. Also, linear regression analyses of *change *in circumferential strain and posterior wall radial strain vs. *change *in RHC parameters were performed. Strain vs. RHC linear regressions were performed on subsets of data before PTE, after PTE and on combined pre- and post-PTE data.

## Results

Right heart catheterization data as well as standard transthoracic echocardiogram data before and after PTE for this group are displayed in Table 1. PTE resulted in statistically significant decreases in both mean PA pressure (44 ± 15 to 29 ± 9 mmHg) and PVR (950 ± 550 to 31 ± 160 [dyne-sec]/cm^5^, p < 0.001 for both). Additionally, there was a statistically significant increase in cardiac output after PTE (3.9 ± 1.0 to 5.0 ± 1.0 L/min, p < 0.001). However, LV ejection fraction (EF), and LV fractional shortening did not change significantly following PTE.

**Table 1:** Patient Data

	Mean ± SD		
Age	51 ± 17		
	Pre-PTE	Post-PTE	p value
Mean PA pressure (mmHg)	44 ± 15	29 ± 9	< 0.001
PVR (dyne-sec)/cm5	950 ± 550	31 ± 160	< 0.001
LVEDD (cm)	4.5 ± 0.7	4.6 ± 0.5	ns
LVESD (cm)	2.7 ± 0.6	2.7 ± 0.5	ns
LV Ejection Fraction (%)	67 ± 8	65 ± 8	ns
Fractional Shortening (%)	40 ± 8	40 ± 7	ns
Cardiac Output (L/min)	3.9 ± 1	5.0 ± 1	< 0.001

LVEDD: left ventricular end-diastolic diameter; LVESD: left ventricular end-systolic diameter;

PA: pulmonary artery; PTE: pulmonary thromboendarterectomy; PVR: pulmonary vascular resistance

Representative sample strain and strain rate curves are shown in Figure 2. Global circumferential strain and SR in the upper boxes is represented by the dotted line. Note the post-PTE radial strain curve in the lower left, demonstrating relative septal hypokinesis. Strain and strain rate data are displayed in Table 2. Circumferential strain decreased by 11% after PTE from -21 ± 3% to -23 ± 4% (p = 0.0002). Posterior radial strain increased by 15% with PTE from 31% ± 8 to 36% ± 11 (p < 0.001). Circumferential strain rate decreased by 7% (from -1.5/s to -1.6/s) and posterior radial SR increased by 5% (from 1.8/s to 1.9/s) after PTE (p = 0.07 and 0.04 respectively). A representative video file of postoperative circumferential strain rate 2D speckle tracking is shown in Additional File 1. Septal radial strain and strain rate did not change significantly following PTE (p = 0.1 and 0.8 respectively).

**Table 2:** Change in Strain and Strain Rate before and after PTE

		Circ. Strain	Circ. SR	Post. Radial Strain	Post. Radial SR	Septal Radial Strain	Septal Radial SR
Mean pre-PTE		-21 ± 3	-1.5 ± 0.3	31 ± 8	1.8 ± 0.3	28 ± 8	1.9 ± 0.5
Mean post-PTE		-23 ± 4	-1.6 ± 0.2	36 ± 11	1.9 ± 0.4	30 ± 11	1.9 ± 0.6
Relative change		-11%	-5%	15%	7%	5%	-0.8%
P-value		< 0.001	0.07	< 0.001	0.04	0.1	0.8

(Mean ± SD). Circ: circumferential; PTE: pulmonary thromboendarterectomy; Post: posterior; SR: strain rate

**Figure 3:**
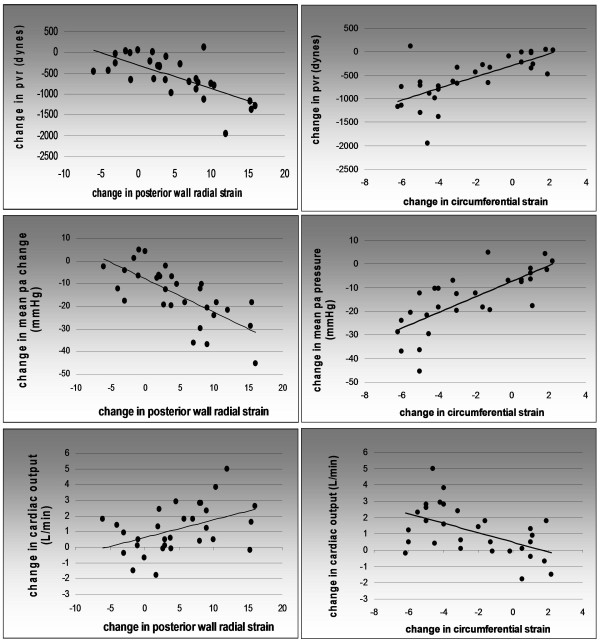
**Linear regression plots of change in posterior wall radial strain (left column) and change in circumferential strain (right column) vs. RHC data**. Measures of linear fit (R values) are displayed in table 3. (Circ: circumferential).

Figure 3 shows scatter plots with corresponding linear regression analyses for circumferential strain and posterior wall radial strain and changes in these parameters vs. RHC data (PVR, mean PA pressure and cardiac output). Linear regression data with r values as a measure of linear fit are displayed in Table 3. Circumferential strain did not correlate well with PVR, mean PA pressure, or cardiac output (r = 0.21, 0.5, 0.17; p = NS for all). Posterior wall radial strain also did not correlate well with PVR, mean PA pressure and cardiac output (r = 0.007, 0.03, and 0.14; p = NS for all). However, *change *in circumferential strain after PTE correlated moderately well with *change *in PVR, mean PA pressure and cardiac output (r = 0.69, 0.76, and 0.51; p < 0.001 for all). Change in posterior wall radial strain correlated moderately well with PVR, mean PA pressure, and cardiac ouput as well (r = 0.7, 0.7, 0.45; p values of < 0.001, < 0.001 and 0.02, respectively).

**Table 3:** Linear Regression

	R (p-value)		
	pre PTE	post PTE	Combined
Circ strain vs PVR	0.1 (0.6)	0.28 (0.2)	0.21 (0.1)
Circ strain vs PAP mean	0.1 (0.6)	0.11 (0.5)	0.5 (0.2)
Circ strain vs CO	0.01 (0.9)	0.33 (.05)	0.17 (0.2)
Radial strain vs PVR	0.08 (0.7)	0.18 (0.3)	0.007 (0.6)
Radial strain vs PAP mean	0.16 (0.4)	0.13 (0.5)	0.03 (0.8)
Radial strain vs CO	0.09 (0.6)	0.18 (0.3)	0.14 (0.3)
Change in circ strain vs change in PVR	NA	NA	0.69 (< 0.001)
Change in circ strain vs change in PAP mean	NA	NA	0.76 (< 0.001)
Change in circ strain vs change in CO	NA	NA	0.51 (0.003)
Change in radial strain vs change in PVR	NA	NA	0.7 (< 0.001)
Change in radial strain vs change in PAP mean	NA	NA	0.7 (< 0.001)
Change in radial strain vs change in CO	NA	NA	0.45 (.02)

Circ: circumferential; CO: cardiac output; PAP: pulmonary artery pressure; PTE: pulmonary thromboendarterectomy; PVR: pulmonary vascular resistance

## Discussion

Chronic thromboembolic pulmonary hypertension has become a reversible disease with the development of PTE surgery. The dramatic and sudden decrease in PAP and PVR after PTE has provided a remarkable model to help study and elucidate RV/LV interactions and biventricular adaptations to severe RV pressure overload. Although the most obvious and striking features of this disease are right ventricular failure along with extreme elevations in PAP and PVR, recent studies have shown that LV diastolic abnormalities are common in CTEPH. Specifically, "impaired relaxation" transmitral Doppler tracings, systolic-dominant pulmonary venous flow patterns, and decreased diastolic tissue Doppler velocities have been observed in CTEPH patients [9]. However, previous studies from our institution suggest that these findings are not indicative of intrinsic LV diastolic dysfunction, as there is a rapid improvement in the Doppler parameters immediately following PTE (including E/A ratio and mitral annular tissue Doppler velocities) [10].

2D speckle tracking is a novel echocardiographic technique that allows digital tracking and quantification of myocardial deformation as a function of time [12]. The deformation can be assessed as both strain (change in length/original length) and strain rate (change in strain/time). Strain and strain rate imaging can assess both circumferential deformation (a negative value in systole, as the circumference of the LV decreases) and radial deformation (a positive value in systole, as the LV wall thickness increases).

In this study of 30 consecutive patients with CTEPH and adequate echo images undergoing PTE with pre- and postoperative RHC, we performed offline 2D speckle measurements of strain and strain rate. PTE resulted in marked improvement in mean PA pressure, PVR, and cardiac output. Additionally, patients demonstrated a statistically significant decrease in circumferential strain (i.e., more circumferential shortening) post-PTE and an increase in posterior wall radial strain (i.e., increased wall thickening). This is likely due to improved left ventricular filling and an increase in LV filling pressure after PTE [9] leading to an improvement in LV performance. The changes could also result from restoration of a more normal LV configuration after PTE.

Septal radial strain, however, did not change significantly post-PTE. Paradoxical

post-operative septal movement and hypokinesis is commonly observed in patients undergoing open heart surgery, and can affect tissue Doppler imaging of the septum as well [17-19]. A recent study, however, has shown normal septal strain rate after bypass graft surgery [20]. The etiology of this regional wall motion abnormality ("post-op septum") is unknown, and may stem from RV dysfunction or a postoperative change in cardiac translation. It is unknown whether the lack of increase in radial septal strain post-PTE in this study is long-lasting, as nearly all patients who undergo PTE at our institution do not live nearby and are unavailable for long term echocardiographic follow-up.

Circumferential as well as posterior wall radial SR did change somewhat after PTE. However, these changes were small compared to the changes in absolute strain (the change in circumferential SR did not reach statistical significance [p = 0.07] and the posterior wall radial SR barely did [p = 0.04]). Like systolic strain, systolic SR is a function of myocardial contractility, preload and afterload: with an increase in LV preload following PTE and, presumably, no change in afterload or LV contractility, one might expect a similar increase in both strain and SR. Why the changes in strain were not mirrored by similar degrees of SR change is unclear. One possibility may stem from the fact that accurate SR analysis requires very precise speckle tracking. As noted in the limitations section, 2D speckle tracking measures strain directly and SR is then calculated by taking the temporal derivative of strain. Hence noise is amplified when determining SR. Although the quality of the echocardiograms was reasonable, there still may have been a degree of "scatter" and artifacts resulting in suboptimal myocardial tracking which was amplified in SR determination.

Radial septal SR did not change significantly with PTE. This is expected considering there was no observed change in radial septal strain with PTE. It is unclear whether the lack of change in septal radial SR post-PTE is long-lasting. One could also ask why LV strain improved without changes in LV dimensions or EF. First, the modified Simpson's method of discs may not apply well to left ventricles that are compressed and distorted in CTEPH, and so the EF calculations may not be completely accurate. Second, we suspect the improvement in strain could be linked to normalization of LV conformation, with a return to a more circular cross-sectional LV shape. Normalization of the LV "eccentricity index" is well documented in patients with CTEPH after PTE [21].

Linear regression analysis of circular strain and posterior wall radial strain vs. hemodynamic parameters revealed several findings. Despite the overall statistically significant change in circumferential and posterior wall radial strain that occurred with PTE, linear correlation of strain values with mean PA pressure, PVR, and cardiac output was poor (Table 3). However, linear regression analysis of the *change *in strain vs. *change *in RHC measurements revealed statistically significant associations. Change in circumferential strain correlated reasonably well with the changes in mean PA pressure, PVR, and cardiac output (r values of 0.69, 0.76, and 0.51 respectively; p < 0.001 for all). Change in posterior wall radial strain also demonstrated a moderate correlation with change in mean PA pressure, PVR, and cardiac output (r values of 0.7, 0.7, and 0.45; p values of < 0.001, < 0.001 and 0.02, respectively). Why did the change in strain correlate more closely with RHC parameters than strain itself? LV strain is influenced by a number of variables, including (but not limited to) longstanding hypertension, cardiac conditioning, coronary artery disease, valvular disease, chamber size, pericardial disease, and volume status [22-27]. Therefore, it is not surprising to find a range of LV strain values for any given degree of pulmonary hypertension. Examining the change in strain following PTE minimizes many of these confounding variables, and may explain the improved correlation between change in strain and RHC variables.

## Limitations

This study has several limitations. First, the 2D speckle tracking software that we utilized requires high quality echocardiographic images and resolute interference patterns for accurate tracking of the myocardium throughout the cardiac cycle. Of the 60 patients evaluated for this study, only 30 had echocardiograms of adequate quality for analysis. As noted in the Methods section, speckle tracking was deemed poor, average, or good by the software's automated tracking algorithm. This determination was based on how well speckle interference patterns could be linked from one frame to the next. Only studies that were deemed good were used in our analysis. One of the reasons for the relatively poor quality of postoperative echocardiograms was the difficulty in obtaining images from patients with surgical dressings and recent thoracotomies. This problem may well limit the clinical feasibility of strain analysis in CTEPH, though technical advances could lead to improved imaging in the future. In addition, strain imaging is not universally available and is used primarily at academic echocardiography centers [28]. We did not evaluate natriuretic peptides before and after PTE [29], and did not address the echo parameters of RV strain, tricuspid annular plane systolic excursion [30] and left atrial size in this study. These are areas of ongoing research at our institution [31,32].

Another limitation was the subjective manual mapping of the region of interest (ROI) over the myocardium. This imprecision was minimized by manual ROI adjustment to achieve good myocardial tracking of all segments as gauged by the Vivid GE software. Additionally, the mean of 3 consecutive strain and SR measurements was used in our statistical analysis. Finally, preoperative transthoracic echocardiography and right heart catheterization were not performed simultaneously in this study, and up to 48 hours elapsed between the two procedures. Because the nature of pulmonary hypertension was chronic in this patient population, we do not believe that simultaneous measurement would have yielded substantially different results. After PTE, the time between initial hemodynamic measurements and echocardiography was longer (mean of 9 days), and it is possible that hemodynamic and Doppler parameters fluctuated during this period. In general, however, patients in this study remained stable after PTE. As with previous studies from our institution, echocardiography was delayed until patients left the surgical intensive care unit and could be positioned properly for examination in the noninvasive cardiac laboratory [9,10,33].

## Conclusions

The hemodynamic changes after PTE were associated with changes of 11% and 15% in LV circumferential and posterior wall radial strain, respectively. The change in circumferential strain was negative (indicating an increase in circumferential contraction), while the change in posterior wall radial strain was positive (indicating an increase in posterior wall systolic thickening after PTE). These changes may stem from improved filling and normalized conformation of the LV post-PTE.

Although there were slight changes in circumferential strain rate and posterior wall radial strain rate post-PTE, these changes were less robust. Changes in strain and SR did not occur in the septum, possibly due to the effects of cardiopulmonary bypass and/or postoperative stunning. Although circumferential strain and posterior wall radial strain correlated poorly with mean PA pressure, PVR and cardiac output, the *change *in LV circumferential strain and posterior wall radial strain correlated well with changes in these hemodynamic parameters.

From a clinical perspective, the changes in circumferential and posterior wall radial LV strain may be useful non-invasive markers of successful PTE (particularly if postoperative TR is minimal and difficult to quantify). In addition, and perhaps more importantly, we believe the findings of this study of LV strain in CTEPH further support the concept [9] that the LV diastolic abnormalities seen in CTEPH are caused in large part by LV underfilling rather than intrinsic LV myocardial changes caused by longstanding right ventricular pressure overload.

## Competing interests

The authors declare that they have no competing interests.

## Authors' contributions

NO performed statistical analysis of the data and wrote the initial manuscript. JB collected the echocardiographic images and analyzed data. AK performed statistical analysis and reviewed the manuscript. WA provided and analyzed hemodynamic data, and reviewed the manuscript. MM performed many of the surgical procedures and participated in the coordination of the study. TW provided echocardiographic data and participated in the design of the study. DB conceived the study, participated in design and data analysis, and revised the initial manuscript. All authors have read and approved the final manuscript.

## Supplementary Material

Additional File 1 **A representative video image of postoperative circumferential LV strain rate 2-D speckle tracking is shown**. Click here for file

## References

[B1] Kearon C Natural history of venous thromboembolism Circulation 2003 107 I22 30 12814982 10.1161/01.CIR.0000078464.82671.78

[B2] Ribeiro A Lindmarker P Johnsson H Juhlin-Dannfelt A Jorfeldt L Pulmonary embolism: one-year follow-up with echocardiography doppler and five-year survival analysis Circulation 1999 16 1325 30 10.1161/01.cir.99.10.1325 10077516

[B3] Becattini C Agnelli G Pesavento R Silingardi M Poggio R Taliani MR Incidence of chronic thromboembolic pulmonary hypertension after a first episode of pulmonary embolism Chest 2006 130 172 5 10.1378/chest.130.1.172 16840398

[B4] Pengo V Lensing AW Prins MH Marchiori A Davidson BL Tiozzo F Incidence of chronic thromboembolic pulmonary hypertension after pulmonary embolism: Thromboembolic Pulmonary Hypertension Study Group N Engl J Med 2004 350 2257 64 10.1056/NEJMoa032274 15163775

[B5] Moser KM Bloor CM Pulmonary vascular lesions occurring in patients with chronic major vessel thromboembolic pulmonary hypertension Chest 1993 103 685 92 10.1378/chest.103.3.685 8449052

[B6] Blauwet LA Edwards WD Tazelaar HD Surgical pathology of pulmonary thromboendarterectomy: a study of 54 cases from 1990 to 2001 Hum Pathol 2003 34 1290 8 10.1016/j.humpath.2003.07.003 14691915

[B7] Fedullo PF Auger WR Channick RN Moser KM Jamieson SW Chronic thromboembolic pulmonary hypertension Clin Chest Med 1995 16 353 74 7656546

[B8] Daniels LB Krummen DE Blanchard DG Echocardiography in pulmonary vascular disease Cardiol Clin 2004 22 383 99 10.1016/j.ccl.2004.04.007 15302359

[B9] Gurudevan SV Malouf PJ Auger WR Waltman TJ Madani M Raisinghani AB DeMaria AN Blanchard DG Abnormal left ventricular diastolic filling in chronic thromboembolic pulmonary hypertension: true diastolic dysfunction or left ventricular underfilling? J Am Coll Cardiol 2007 49 1334 9 10.1016/j.jacc.2007.01.028 17394966

[B10] Mahmud E Raisinghani A Hassankhani A Sadeghi HM Strachan GM Auger W DeMaria AN Blanchard DG Correlation of left ventricular diastolic filling characteristics with right ventricular overload and pulmonary artery pressure in chronic thromboembolic pulmonary hypertension J Am Coll Cardiol 2002 40 318 24 10.1016/S0735-1097(02)01959-9 12106938

[B11] Menzel T Wagner S Kramm T Mohr-Kahaly S Mayer E Braeuninger S Pathophysiology of impaired right and left ventricular function in chronic embolic pulmonary hypertension: changes after pulmonary thromboendarterectomy Chest 2000 118 897 903 10.1378/chest.118.4.897 11035654

[B12] Perk G Tunick PA Kronzon I Non-Doppler two-dimensional strain imaging by echocardiography--from technical considerations to clinical applications J Am Soc Echocardiogr 2007 20 234 43 10.1016/j.echo.2006.08.023 17336748

[B13] Thistlethwaite PA Kaneko K Madani MM Jamieson SW Technique and outcomes of pulmonary endarterectomy surgery Ann Thorac Cardiovasc Surg 2008 14 274 82 18989242

[B14] Schiller NB Shah PM Crawford M Recommendations for quantitation of the left ventricle by two-dimensional echocardiography. American Society of Echocardiography Committee on Standards, Subcommittee on Quantitation of Two-Dimensional Echocardiograms J Am Soc Echocardiogr 1989 2 358 67 2698218 10.1016/s0894-7317(89)80014-8

[B15] Feigenbaum H The Echocardiographic Examination Feigenbaum's Echocardiography 2005 Philadelphia, PA: Lippincott Williams & Wilkins Publications 126 127

[B16] Wessa P Free Statistics Software, Office for Research Development and Education 2009 1.1.23-r4 http://www.wessa.net/

[B17] Lehmann KG Lee FA McKenzie WB Onset of altered interventricular septal motion during cardiac surgery. Assessment by continuous intraoperative transesophageal echocardiography Circulation 1990 82 1325 34 2401066 10.1161/01.cir.82.4.1325

[B18] Wranne B Pinto FJ Siegel LC Abnormal postoperative interventricular motion: new intraoperative transesophageal echocardiographic evidence supports a novel hypothesis Am Heart J 1993 126 161 7 10.1016/S0002-8703(07)80024-X 8322660

[B19] Malouf PJ Madani MM Gurudevan SV Waltman TJ Raisinghani A DeMaria AN Blanchard DG Assessment of diastolic function with tissue Doppler imaging after cardiac surgery: Effects of the "postoperative septum" in on-pump vs. off-pump procedures J Am Soc Echocardiogr 2006 19 464 7 10.1016/j.echo.2005.12.001 16581488

[B20] Toyoda T Akasaki T Watanabe N Evaluation of abnormal motion of interventricular septum after coronary artery bypass grafting operation: assessment by ultrasonic strain rate imaging J Am Soc Echocardiogr 2004 17 711 6 10.1016/j.echo.2004.03.033 15220894

[B21] Dittrich HC Nicod PH Chow LC Chappuis FP Moser KM Peterson KL Early changes of right heart geometry after pulmonary thromboendarterectomy J Am Coll Cardiol 1988 11 937 43 10.1016/S0735-1097(98)90049-3 3356839

[B22] Yan YT Wenzelburger F Lee E Heatlie G Leyva F Patel K The pathophysiology of heart failure with normal ejection fraction exercise echocardiography reveals complex abnormalities of both systolic and diastolic ventricular function involving torsion, untwist, and longitudinal motion J Am Coll Cardiol 2009 30 36 46 10.1016/j.jacc.2009.03.037 19555838

[B23] Marwick TH Leano RL Brown J Sun JP Hoffmann R Lysyansky P Myocardial strain measurement with 2-dimensional speckle-tracking echocardiography: definition of normal range J Am Coll Cardiol Img 2009 2 80 4 10.1016/j.jcmg.2007.12.007 19356538

[B24] Nesbitt GC Mankad S Strain and strain rate imaging in cardiomyopathy Echocardiography 2009 26 337 44 10.1111/j.1540-8175.2008.00867.x 19291019

[B25] Choi JO Shin DH Cho SW Song YB Kim JH Kim YG Effect of preload on left ventricular longitudinal strain by 2D speckle tracking Echocardiography 2008 25 873 9 10.1111/j.1540-8175.2008.00707.x 18986415

[B26] Rösner A Bijnens B Hansen M How OJ Aarsaether E Müller S Left ventricular size determines tissue Doppler-derived longitudinal strain and strain rate Eur J Echocardiogr 2009 10 271 7 10.1093/ejechocard/jen230 18827033

[B27] Kim H Cho HO Cho YK Nam CW Han SW Hur SH Relationship between early diastolic strain rate imaging and left ventricular geometric patterns in hypertensive patients Heart Vessels 2008 23 271 8 10.1007/s00380-008-1042-0 18649058

[B28] Citro R Bossone E Kuersten B Gregario G Salustri A Tissue Doppler and strain imaging: anything left in the echo lab? Cardiovascular Ultrasound 2008 6 54 10.1186/1476-7120-6-54 18973677 PMC2583989

[B29] Suntharalingam J Goldsmith K Toshner M Doughty N Sheares KK Hughes R Role of NT-pro BNP and 6MWD in chronic thromboembolic pulmonary hypertension Respir Med 2007 101 2254 62 10.1016/j.rmed.2007.06.027 17706409

[B30] Giusca S Dambrauskaite V Scheurwegs C D'hooge J Claus P Herbots L Deformation imaging describes right ventricular function better than longitudinal displacement of the tricuspid ring Heart 2010 96 281 288 10.1136/hrt.2009.171728 19720609

[B31] Olson N Brown JP Kahn AM Auger WR Madani MM Kopelnik A Kolski B Waltman TJ Blanchard DG Right ventricular basal strain and strain rate by 2D speckle tracking in chronic thromboembolic pulmonary hypertension before and after pulmonary thromboendarterectomy (abstr) J Am Soc Echocardiogr 2009 22 591

[B32] Parise C Kimura B Daniels L Brown J Kahn A Waltman T Blanchard DG Aortic excursion in chronic thromboembolic pulmonary hypertension (abstr) CHEST 2008 134 s47004 10.1111/echo.12252 23710685

[B33] Blanchard DG Malouf PJ Gurudevan SV Auger WR Madani MM Thistlethwaite P Waltman TJ Daniels LB Raisinghani A DeMaria AN Utility of right ventricular Tei index in the noninvasive evaluation of chronic thromboembolic pulmonary hypertension before and after pulmonary thromboendarterectomy J Am Coll Cardiol Img 2009 2 143 9 10.1016/j.jcmg.2008.10.012 19356547

